# Brainstem and spinal cord MRI identifies altered sensorimotor pathways post-stroke

**DOI:** 10.1038/s41467-019-11244-3

**Published:** 2019-08-06

**Authors:** Haleh Karbasforoushan, Julien Cohen-Adad, Julius P. A. Dewald

**Affiliations:** 1 Interdepartmental Neuroscience Program, Feinberg School of Medicine, Northwestern University, Chicago, 60611 IL USA; 2 Department of Physical Therapy and Human Movement Sciences, Feinberg School of Medicine, Northwestern University, Chicago, 60611 IL USA; 3 NeuroPoly Lab, Institute of Biomedical Engineering, Polytechnique Montréal, Montréal, H3T 1J4 QC Canada; 4 Functional Neuroimaging Unit, CRIUGM, University of Montreal, Montreal, H3T 1J4 QC Canada; 5 Department of Biomedical Engineering, McCormick School of Engineering, Northwestern University, Evanston, 60208 IL USA

**Keywords:** Magnetic resonance imaging, Spinal cord, Stroke

## Abstract

Damage to the corticospinal tract is widely studied following unilateral subcortical stroke, whereas less is known about changes to other sensorimotor pathways. This may be due to the fact that many studies investigated morphological changes in the brain, where the majority of descending and ascending brain pathways are overlapping, and did not investigate the brainstem where they separate. Moreover, these pathways continue passing through separate regions in the spinal cord. Here, using a high-resolution structural MRI of both the brainstem and the cervical spinal cord, we were able to identify a number of microstructurally altered pathways, in addition to the corticospinal tract, post stroke. Moreover, decreases in ipsi-lesional corticospinal tract integrity and increases in contra-lesional medial reticulospinal tract integrity were correlated with motor impairment severity in individuals with stroke.

## Abstract (web summary)

There are few studies of structural changes in ascending and descending sensorimotor pathways after stroke, beyond the corticospinal tract, in the brain. Here the authors identify changes in white matter structure in brainstem and spinal cord following stroke, and show its relationship to motor impairment.

## Introduction

Subcortical unilateral strokes affecting the internal capsule or basal ganglia are the most common of all strokes and usually result in hemiparesis of the contralateral arm and leg. Previous human brain imaging studies of unilateral subcortical stroke have frequently reported damage to the corticospinal tract^1–4^. Cross-sectional studies also reported this damage to be associated with greater motor deficits as well as worse motor recovery in these individuals^5–8^. However, less is known about other altered sensorimotor pathways post unilateral stroke and their role in motor impairments. An important reason for this lack of knowledge is that previous studies have only investigated the morphological changes in the brain, where the majority of descending and ascending brain pathways (e.g., corticospinal tract, cortico-bulbospinal tracts, dorsal column medial lemniscus) mostly overlap and are not distinguishable with currently available imaging techniques. Sensorimotor pathways, in fact, delineate from each other in the brainstem^9–12^. Moreover, these tracts continue travelling through separate regions in the spinal cord^9,10^.

It is also noteworthy to mention that numerous functional neuroimaging studies of stroke have reported increased neural activity in motor cortices of both lesioned and non-lesioned hemisphere during hand/arm movement^13–16^. They have also reported that the more severe the impairment, the greater the activity in the contralesional hemisphere compared to the ipsilesional (i.e., a shift of activity to non-lesioned hemisphere)^17–23^. However, it is still unclear what descending motor pathway allows the contralesional motor cortex to control the ipsilateral paretic arm, although one proposed idea from human and animal studies suggests^24–28^ brainstem ipsilaterally projecting motor pathways (i.e., reticulospinal tracts or vestibulospinal tracts) may play this role.

Based on magnetic resonance imaging (MRI), diffusion tensor imaging (DTI) can probe white matter microstructure by providing information on the direction and degree of water diffusivity in white matter tracts^29^. The degree of anisotropy (fractional anisotropy: FA) of water in the white matter tracts reflects their level of integrity^30,31^. Evidence from human brain imaging studies suggests that white matter integrity could change with experience. For example, damage or degeneration to a tract is shown to be associated with a decrease in its white matter integrity (FA)^1–3,32–34^. Likewise, training-induced or experience-dependent increases in white matter integrity have also been reported in humans^35–37^. Moreover, recent developments in MRI of the spinal cord have opened new doors to further studies of the human nervous system^38–40^. High-resolution DTI of cervical spinal cord and novel analyses approaches provide means of investigating the spinal cord white matter tracts. DTI’s ability to determine long-term changes in white matter structure and its recent developments in the cervical spinal cord^38–40^ makes this an excellent method for investigating altered sensorimotor pathways in the brainstem and spinal cord in individuals with chronic stroke.

To this end, we used high-resolution structural MRI of the brainstem and cervical spinal cord and unbiased voxel-wise analyses to identify all altered sensorimotor pathways post unilateral subcortical stroke. Subsequently, the link between microstructural changes in pathways and paretic upper limb motor impairments (Fugl–Meyer Assessment (FMA)) was examined in the stroke participants. It was hypothesized that individuals with stroke would show reduced white matter integrity in a number of sensorimotor pathways travelling through subcortical regions of lesioned hemisphere, in addition to the corticospinal tract. Furthermore, an increased white matter integrity in some brainstem ipsilaterally projecting pathways from the non-lesioned hemisphere was expected. Our results indicate a significant decrease of white matter integrity in corticospinal tract, lateral and medial reticulospinal tracts, descending medial longitudinal fasciculus, tectospinal tract, and cuneate and gracile fasciculi related to the lesioned hemisphere. Furthermore, the results from brainstem and cervical spinal cord DTI analyses indicate a significant increase in the white matter integrity of medial reticulospinal tract at the side of contralesional hemisphere, which projects ipsilaterally to the paretic (contralesional) limbs. The decreased white matter integrity of ipsilesional corticospinal tract and increased white matter integrity of contralesional medial reticulospinal tract are correlated with upper limb impairment severity in individuals with stroke.

## Results

### Demographics

Of the subjects recruited, five stroke participants and one healthy control were excluded from the study due to brainstem, bilateral or cortical lesions, or poor-quality image. Therefore, the final sample consisted of 31 individuals with stroke and 31 healthy controls. Demographics of two groups are presented in Table 1. The stroke group had more African-American participants than the controls group (*x*^2^ = 8.72, *p* = 0.01). The two groups were well matched on age and gender. The lesion map of individuals with stroke is presented in Supplementary Fig. 1.

**Table 1:** Demographic characteristics

Variable	Controls		Stroke		*x* ^2^	df	*p* Value
*N*	31		31				
Gender (male:female)	18:13		20:11		0.27	1	0.602
Ethnicity (White:AA:Other)	26:4:1		15:13:3		8.72	4	0.012
Affected side of body (left:right)	—		15:16		—	1	—

	Mean	SD	Mean	SD	*t*	df	*p* Value
Age	61.61	9.45	59.83	8.97	0.76	60	0.452
Years since onset	—	—	11.48	7.65	—	30	—
Fugl–Meyer^a^	—	—	31.00	15.78	—	29	—

^a^Fugl–Meyer score of one individual with stroke is missing

### Brainstem white matter integrity in stroke

**Fig. 1:**
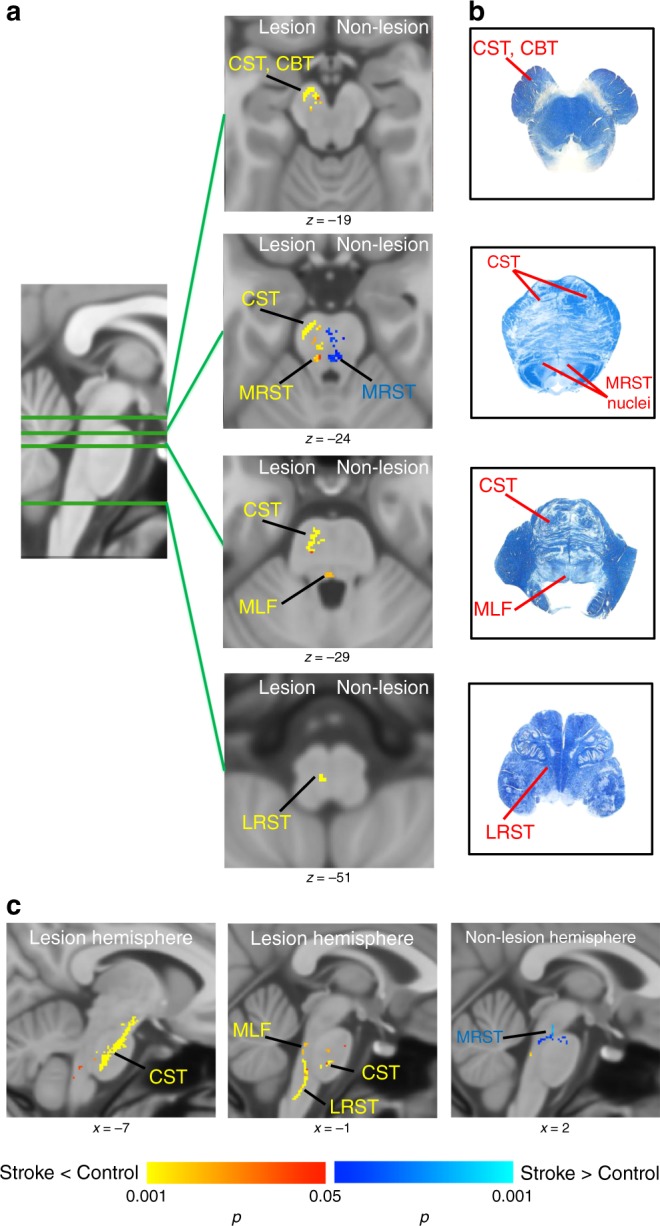
Tracts with white matter integrity changes in brainstem in individuals with stroke compared to controls. **a** Axial view of the tracts with significant decrease (red-yellow) or increase (blue) in white matter integrity in individuals with stroke compared to controls. Corticospinal tract (CST), corticobulbar tract (CBT), medical reticulospinal tract (MRST), lateral reticulospinal tract (LRST), and medial longitudinal fasciculus (MLF) of the lesioned hemisphere showed significant decreased white matter integrity in individuals with stroke compared to controls. Medial reticulospinal tract (MRST) of non-lesioned hemisphere showed significant increased white matter integrity in individuals with stroke. **b** Brainstem atlas adapted from Gray’s anatomy and Haines’ neuroanatomy with modification for simplicity, showing the location of tracts. **c** Sagittal view of the tracts with significant decrease (red-yellow) or increase (blue). (Statistical test: two-sample *t* test with non-parametric 50,000 permutation. Using the threshold-free cluster enhancement with corrected *p* < 0.05)

Tracts with significant white matter integrity changes in the brainstem of individuals with stroke compared to controls are presented in Fig. 1 (threshold-free cluster enhancement *p* = 0.05). In the brainstem at the side of lesioned hemisphere, we observed decreased white matter integrity in corticospinal and bulbospinal tracts, including medial reticulospinal, lateral reticulospinal, and medial longitudinal fasciculus (i.e., descending medial vestibulospinal tract) in individuals with stroke compared to controls (Fig. 1, tracts in red-yellow color). Consistent with our hypotheses, at the side of the non-lesioned hemisphere, an increased white matter integrity in individuals with stroke was found in the medial reticulospinal tract, which receives projections from non-lesioned motor cortices (Fig. 1, tracts in blue-light blue color). The locations of sensorimotor tracts in the brainstem were determined with the help of Gray’s anatomy^9^, Haines’ neuroanatomy^10^, and Paxinos’ brainstem anatomy^11,41^. Fig. 1b shows the location of tracts on the brainstem atlas, focusing on the sensorimotor pathways under investigation, for simplicity.

**Fig. 2:**
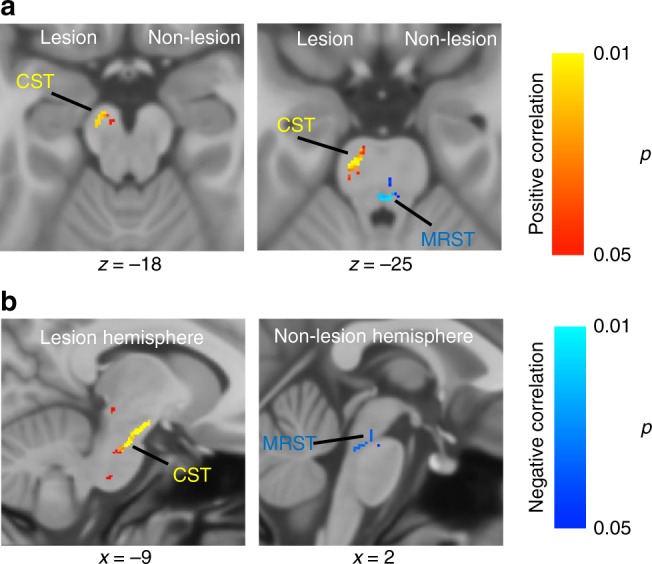
Tracts with significant correlation between white matter integrity changes and motor impairment. **a** Axial view of the tract with positive correlation (red-yellow) and negative correlation (blue) between white matter integrity and motor impairment (Fugl–Meyer assessment, FMA) in individuals with stroke. Corticospinal tract (CST) of the lesion hemisphere showed significant positive correlation with impaired motor performance in individuals with stroke. Medial reticulospinal tract (MRST) at the side of non-lesioned hemisphere showed significant negative correlation with impaired motor performance in individuals with stroke. **b** Sagittal view of the tracts with significant positive (red-yellow) or negative (blue) correlation between white matter integrity and impaired motor performance in individuals with stroke. (Statistical test: within-group voxel-wise correlation analysis, using the threshold-free cluster enhancement and *p* < 0.05)

We then tested for any associations between these white matter alterations and clinical motor assessments in the paretic upper limb (Fugl–Meyer Motor Assessment score). Figure 2 illustrates the tracts with significant correlation between white matter integrity changes and motor impairment in individuals with stroke. Decreases in corticospinal tract integrity at the lesioned hemisphere and increases in medial reticulospinal tract integrity at the non-lesioned hemisphere in individuals with stroke were shown to be significantly correlated with their motor impairment severity (Fig. 2).

### Spinal cord white matter integrity in stroke

**Fig. 3:**
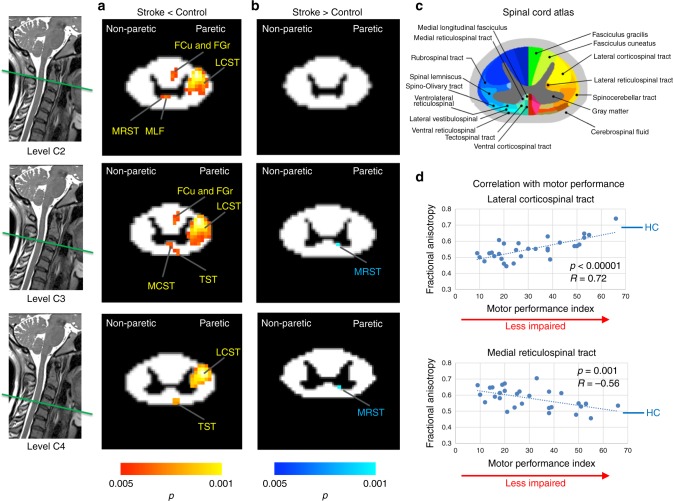
White matter integrity changes in spinal cord of individuals with stroke compared to controls. **a** Tracts with significant decrease (red-yellow) in white matter integrity (fractional anisotropy (FA)). When compared to controls, individuals with stroke had significant decrease in white matter integrity of lateral corticospinal tract (LCST), cuneate fasciculus (FCu), gracile fasciculus (FGr), and tectospinal tract (TST) of the paretic side. These individuals also showed decreased white matter integrity in medial reticulospinal tract (MRST), medial longitudinal fasciculus (MLF), and medial corticospinal tract (MCST) of non-paretic side, which project ipsilaterally from the lesioned hemisphere. **b** Tracts with significant increase (blue) in white matter integrity. Medial reticulospinal tract of paretic side, which projects from non-lesioned motor cortices, had significant increased white matter integrity in individuals with stroke compared to controls. (Statistical test: between-group two-sample *t* test. Voxel-wise *p* < 0.005 was applied and the cluster-level corrected at *p* = 0.05.) **c** High-resolution spinal cord white matter atlas derived from the Gray’s Anatomy Atlas, adapted from ref. ^42^. **d** Correlation of significant white matter integrity changes in individuals with stroke with their motor impairment level. Decreases in lateral corticospinal tract in individuals with stroke had significant positive correlation with their impaired motor performance. Increases in medial reticulospinal tract in individuals with stroke showed a negative correlation with their impaired motor performance. Paretic refers to the side of non-lesioned hemisphere (contralesional). Blue line with abbreviation HC indicates the mean of FA value in healthy controls for lateral corticospinal tract (equal to 0.68) and medial reticulospinal tract (equal to 0.49). Fugl–Meyer Assessment (FMA) score is missing for one participant. Source data are provided as a Source Data file

Figure 1 shows the tracts with significant white matter integrity changes in cervical spinal cord in individuals with stroke compared to the controls. The results from the cervical spinal cord analysis indicated a significant decrease in white matter integrity of lateral corticospinal tract, tectospinal tract, and cuneate and gracile fasciculi at the paretic (contralesional) side in individuals with stroke. The stroke participants also demonstrated a significantly decreased white matter integrity in a few smaller clusters, which, to our current knowledge of spinal cord pathways locations, appear to belong to the medial reticulospinal tract, medial longitudinal fasciculus, and medial corticospinal tract at the non-paretic side of body. Of note, these pathways project ipsilaterally from the lesioned hemisphere (Fig. 3a, in red-yellow color). Consistent with results from the brainstem analyses, patients showed a significant increase in white matter integrity in a small cluster that, to our current knowledge of spinal cord pathways, belongs to the medial reticulospinal tract on paretic (contralesional) side, which projects ipsilaterally from the non-lesioned hemisphere (Fig. 3b, in blue-light blue color). All resulted tracts are thresholded with a voxel-wise *p* value of 0.005 and corrected for cluster size with a *p* value of 0.05. Figure 3c illustrates the high-resolution spinal cord white matter atlas derived from the Gray’s anatomy atlas, adapted from ref. ^42^.

Testing for any associations between these white matter differences in cervical spinal cord and clinical assessments of motor impairment in individuals with stroke, we found that the decreased white matter integrity of lateral corticospinal tract and increased white matter integrity of medial reticulospinal tract were correlated with motor impairment severity in these individuals (Fig. 3d). In other words, a greater severity of motor impairment was correlated with a lower white matter integrity of the lesioned lateral corticospinal tract and higher white matter integrity of the contralesional medial reticulospinal tract.

## Discussion

Previous human brain imaging studies of unilateral subcortical stroke have frequently reported the damage to the corticospinal tract and its link with greater motor impairment^1–8^. While there are some other sensorimotor pathways passing through lesioned subcortical regions as well, less is known about other altered sensorimotor tracts post unilateral stroke and their role in motor impairment. An important reason for this lack of knowledge is that main descending and ascending brain pathways mostly overlap in the brain and delineate from each other in the brainstem^9–12^, while the previous studies have only investigated the morphological changes in the brain. Additionally, these tracts also continue to pass through separate regions in the spinal cord^9,10^.

In this study, we used high-resolution structural MRI (DTI) of the brainstem and cervical spinal cord and unbiased voxel-wise analyses that, to the best of our knowledge, constitutes the only approach to identify all the sensorimotor pathways with white matter changes after unilateral subcortical stroke. Our results from the brainstem analyses show that the corticospinal and bulbospinal tracts (i.e., medial and lateral reticulospinal tracts and descending medial vestibulospinal tracts) at the side of lesioned hemisphere had decreased white matter integrity in individuals with stroke compared to controls. It is worth noting that these corticospinal and cortico-bulbospinal pathways pass together through the internal capsule—the lesioned region.

Our findings from spinal cord analyses further revealed decreased white matter integrity in cuneate fasciculus, gracile fasciculus, and tectospinal tract of paretic (contralesional) side, which are also linked with the lesioned hemisphere. Decreased white matter integrity of gracile and cuneate fasciculi clearly provides support to previous studies that indicate impairments in tactile and proprioceptive sensations post unilateral stroke^43–46^.

Last but not least, the results from both brainstem and cervical spinal cord analyses indicated increased white matter integrity of the medial reticulospinal tract, which gets corticobulbar projections from non-lesioned hemisphere motor cortices and projects ipsilaterally from reticular nuclei to spinal cord^9–12^. The combination of decreased white matter integrity in a number of sensorimotor pathways of the lesioned hemisphere and increased white matter integrity of the medial reticulospinal tract of the non-lesioned hemisphere is the most striking aspect of our results. This may provide an explanation for a large number of previous studies reporting an increase in activation of motor cortices on non-lesioned hemisphere during use of paretic arm in individuals with stroke^13–15^. More interestingly, we found that decreases in corticospinal tract integrity at the lesioned hemisphere and increases in medial reticulospinal tract integrity at the non-lesioned hemisphere in stroke individuals were significantly correlated with their motor impairment. These results imply that motor impairments post unilateral stroke are associated with the damage to the corticospinal tract of lesioned hemisphere and also with increased reliance on the medial reticulospinal tract of non-lesion hemisphere. The positive correlation between increased white matter integrity of contralesional medial reticulospinal tract and impairment severity in these individuals is consistent with previous reports of correlation between impairment severity and greater shift of activity to contralesional hemisphere^17–23^. These results have implications for rehabilitation interventions post unilateral stroke. Next, we need to further investigate the role of medial reticulospinal tract in motor control through functional experiments; this knowledge will help us optimize the rehabilitation strategies to work with the innate capabilities of this pathway.

There are a couple of limitations of the current investigation that merit consideration when interpreting the results. First, with regard to the spinal cord MRI spatial resolution, although our acquisition setup comprises the current state of the art in the field of neuroimaging, there is still room for its improvement with the next generation of MRI scanners with better gradient systems. Second, even though we have taken a great care using the most detailed currently known maps of the brainstem and spinal cord tracts from a number of atlases, future improvement in imaging approaches may further inform the correctness of the location of pathways in the human brainstem and spinal cord^47^. Follow-up investigations in the next decades with more advanced MRI scanners and improved knowledge of maps of brainstem and spinal cord tracts may further help refine the anatomical specificity of white matter tracts affected post unilateral stroke.

## Methods

### Participants

Thirty-six individuals with chronic hemiparetic unilateral stroke in internal capsule (ischemic or hemorrhagic) and 32 age-matched, gender-matched healthy controls with no neurological or movement disorder were recruited to participate in this study. Stroke subjects were mildly to severely impaired and all sustained a unilateral brain lesion at least 3 years prior to participation in the study. Individuals with stroke were recruited through the Northwestern Clinical Neuroscience Research Registry, Ischemic Stroke Registry, and approved flyers. This study was approved by the Institutional Review Board of Northwestern University, and all subjects provided written informed consent prior to participation.

### Clinical assessment

For each stroke individual, upper extremity motor assessment (FMA^48^) was performed by a licensed physical therapist. FMA is a performance-based impairment index assessing motor functioning, joint functioning, sensation, and balance in upper limb. Higher values indicate higher motor abilities (less impairment) in these individuals.

### Scanning parameters

Brainstem and cervical spinal cord (C2–C5) MRI scans were acquired at Northwestern University Center for Translational Imaging on a 3 T Siemens Prisma scanner with a 64-channel head coil. Brainstem T1-weighted anatomical scans were acquired using an MPRAGE (magnetization-prepared rapid gradient echo) sequence with voxel size = 0.8 × 0.8 × 0.8 mm, repetition time (TR) = 9.9 ms, echo time (TE) = 4.6 ms, flip angle = 2°, and field of view (FOV) = 256 mm. Brainstem diffusion-weighted images (DWIs) were collected using spin-echo echo-planner imaging with the following parameters: voxel size = 1.5 isotropic, TR = 3620 ms, TE = 68.4 ms, matrix size = 150 × 150, FOV = 222 × 222 mm^2^, slice thickness = 1.5 mm, interslice gap = 0 mm, and number of slices covering whole brain and brainstem = 108. The sequence consisted of diffusion weighting of 1000 s/mm^2^ in 60 different directions and 8 scans with no diffusion weighting (*b* = 0 s/mm^2^).

Cervical spinal cord T2-weighted anatomical scans were collected with the resolution = 0.8 × 0.8 × 0.8, TR = 1500 ms, TE = 100 ms, FOV = 256 × 256 mm^2^, number of slices = 64, and scan duration = 4:35 min. Cervical spinal cord DWI scans were collected from level C2 to C5 with four acquisitions averaged offline with the following parameters: in-plane resolution = 0.8 × 0.8, TR = ~600 ms (depends on heart beat), TE = 61 ms, slice thickness = 5 mm, interslice gap = 0 mm, FOV = 86 mm^2^, number of slices = 15, *b* value = 1000 in 30 directions, 4 images with *b* = 0, and scan duration = 6:35 min. For a better gray matter–white matter segmentation of spinal cord, we also collected a T2*-weighted scan that used multi-echo recombined gradient echo with three echoes at 5, 10, and 20 ms, TR = 769 ms, in-plane resolution = 0.4 × 0.4 mm^2^, slice thickness = 5 mm, number of slices = 15, and the scan duration = 5:11 min.

### Brainstem DTI data preprocessing

The DWIs were pre-processed using the FMRIB Software Library version 5.0.11 (FSL: Oxford Centre for Functional MRI of the Brain, UK; http://www.fmrib.ox.ac.uk/fsl/)^49,50^. Raw DTI data were corrected for eddy current distortions using the Eddy Current Correction tool and for motion using the McFlirt Motion Correction tool. The FMRIB Brain Extraction Tool^51^ was then used for skull stripping. Diffusion tensors were fitted at each voxel and FA images were created using the DTIFIT tool of FMRIB FDT toolbox version 5.0^49^.

### Brainstem tract-based spatial statistics analysis

Majority of stroke participants had lesion in the left hemisphere. For those individuals with lesions in the right hemisphere, FA maps were flipped so that all subjects had lesions in the left hemisphere for group analysis. Voxel-wise statistical analysis of the DTI FA images was carried out using tract-based spatial statistics (TBSS^52^) in FSL. First, all subjects’ FA images were aligned to the standard 1 × 1 × 1 mm^3^ MNI152 template in FSL, using the non-linear registration tool (FNIRT). Next, the mean FA image was created and thinned to create a mean FA skeleton representing the centers of all tracts, using a threshold of 0.25. Each subject’s aligned FA map was then projected onto this skeleton resulting in each subject’s skeletonized FA image, and then masked by brainstem standard template to limit the analysis to the brainstem.

Group differences in voxel-wise FA were examined by entering each subjects’ brainstem skeletonized FA into a general linear model (two-sample *t* test) design matrix with non-parametric permutation testing, using the Randomize tool in FSL (50,000 permutations). The results were thresholded at *p* = 0.05 (corrected), using the threshold-free cluster enhancement option to find clusters without setting an initial cluster level^53^.

To further test any association between these group differences and motor impairment in individuals with stroke, we ran a within-group voxel-wise correlation between stroke participants’ brainstem skeletonized FA masked by group differences and each subject’s clinical assessment of performance. Results were corrected at *p* = 0.05 using the threshold-free cluster enhancement option to find the clusters with significant positive or negative correlation with motor impairment level in stroke individuals.

### Spinal cord data preprocessing

All spinal cord images were visually inspected and excluded if motion, low signal, or artifacts were present. Data preprocessing was done using spinal cord toolbox version 3.2.2 (SCT; https://www.nitrc.org/projects/sct/)^54^. Automatic spinal cord segmentation was performed on T2WI, T2*WI, and DWI scans^55^. Images were nonlinearly registered to the MNI-Poly-AMU template/atlas in SCT^42^. T2*WI image, which has a strong contrast between gray matter and white matter, was further analyzed with automatic segmentation of gray matter and white matter, and was used to refine the registrations to the template.

DTI data were corrected for motion and registered to the template. FA maps were then created for each subject and registered to the template, using the same refined registration matrix.

### Spinal cord DTI voxel-wise statistical analyses

For those individuals with lesions in the right hemisphere (left paretic side), spinal cord FA maps were flipped so that all subjects included in the group analysis had the paretic side presented on right. Statistical analysis of spinal cord DTI data was conducted using SPM12 (Statistical Parametric Mapping; https://www.fil.ion.ucl.ac.uk/spm/software/spm12/) software on MATLAB 2018a (https://www.mathworks.com/products/matlab.html). Statistical analysis was done by entering the individual subject FA maps into between-group two-sample *t* test analyses. Since some subjects had a lower signal-to-noise ratio at the vertebral level C5, the between-group analysis was limited to the C2–C4 level. Voxel-wise *p* = 0.005 was applied and the cluster level corrected at *p* = 0.05.

### Reporting summary

Further information on research design is available in the Nature Research Reporting Summary linked to this article.

## Supplementary information

Supplementary Information

Reporting Summary

Source Data

Source Data

## Data Availability

The data underlying the findings of this study are available from the corresponding author upon reasonable request. The source data underlying Fig. 3d are provided as a Source Data file.
