# Author Correction: Brainstem and spinal cord MRI identifies altered sensorimotor pathways post-stroke

**DOI:** 10.1038/s41467-020-17024-8

**Published:** 2020-07-06

**Authors:** Haleh Karbasforoushan, Julien Cohen-Adad, Julius P. A. Dewald

**Affiliations:** 1 Interdepartmental Neuroscience Program, Feinberg School of Medicine, Northwestern University, Chicago, 60611 IL USA; 2 Department of Physical Therapy and Human Movement Sciences, Feinberg School of Medicine, Northwestern University, Chicago, 60611 IL USA; 3 NeuroPoly Lab, Institute of Biomedical Engineering, Polytechnique Montréal, Montréal, H3T 1J4 QC Canada; 4 Functional Neuroimaging Unit, CRIUGM, University of Montreal, Montreal, H3T 1J4 QC Canada; 5 Department of Biomedical Engineering, McCormick School of Engineering, Northwestern University, Evanston, 60208 IL USA

**Keywords:** Magnetic resonance imaging, Spinal cord, Stroke

Correction to: *Nature Communications* 10.1038/s41467-019-11244-3, published online 06 August 2019.

The original version of this Article omitted references to three previous relevant studies: Owen, M., Ingo, C. & Dewald, J. Upper extremity motor impairments and microstructural changes in bulbospinal pathways in chronic hemiparetic stroke. *Front. Neurol.* **8**, 257 (2017); Rüber, T., Schlaug, G. & Lindenberg, R. Compensatory role of the cortico-rubro-spinal tract in motor recovery after stroke. *Neurology* **79**, 515–522 (2012) and Takenobu, Y. et al. Motor recovery and microstructural change in rubro-spinal tract in subcortical stroke. *NeuroImage Clin*. **4**, 201–208 (2014). These have been included as references 43–45 in a new paragraph in the Discussion, which reads “Of the limited number studies that have looked at brainstem morphological changes, it is noteworthy to mention that a previous stroke study, using diffusion tensor imaging with a smaller sample size, less restricted inclusion criteria in terms of lesion location, and different imaging protocols, did not report any significant increased FA in individuals with stroke compared to controls in voxel-wise and ROI analyses^43^. Two other studies reported an increased FA in the rubro-spinal tract in a smaller sample size of individuals with more acute stroke^44,^^45^.”

The Article contained an error in a sentence in the Introduction, which included the phrase “the previous studies have only investigated the morphological changes in the brain”. The corrected sentence in the Introduction reads “An important reason for this lack of knowledge is that most previous studies have only investigated the morphological changes in the brain, where the majority of descending and ascending brain pathways (e.g., corticospinal tract, cortico-bulbospinal tracts, dorsal column medial lemniscus) mostly overlap and are not distinguishable with currently available imaging techniques”.

The Article also contained an error in the Discussion, which contained the phrase “while the previous studies have only investigated the morphological changes in the brain”. The corrected sentence in the Discussion reads “An important reason for this lack of knowledge is that main descending and ascending brain pathways mostly overlap in the brain and delineate from each other in the brainstem^9–12^, while most previous studies have only investigated the morphological changes in the brain.”

These have been corrected in the PDF and HTML versions of the Article.

