# Supplementary material for: Brainstem and spinal cord MRI identifies altered sensorimotor pathways post-stroke
Source: Nat Commun. 2019 Aug 6;10:3524. doi: 10.1038/s41467-019-11244-3 (PMC6684621; doi:10.1038/s41467-019-11244-3)
Supplement: Supplementary file 1 — Supplementary Information [file 41467_2019_11244_MOESM1_ESM.pdf]

Brainstem and spinal cord MRI identifies altered sensorimotor pathways post-stroke

Karbasforoushan et al.

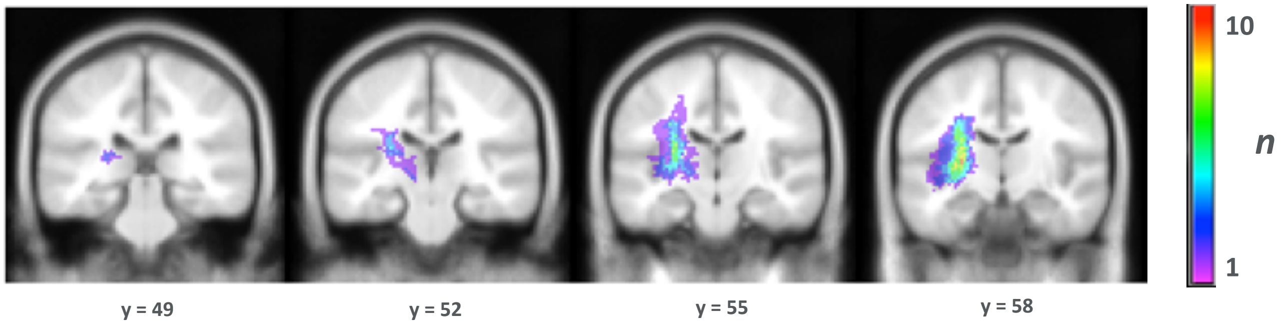

Supplementary Figure 1. Stroke lesion overlap map. Coronal view of the stroke lesion overlap map for the group of 31 individuals with stroke. Majority of stroke participants had lesion in the left hemisphere. For those individuals with lesions in the right hemisphere, lesion maps were flipped so that all subjects had lesions presented in the left hemisphere. y provides the coronal slide coordinate. *n* indicates the number of participants with a lesion in a particular location.
