# Supplementary material for: Brainstem and spinal cord MRI identifies altered sensorimotor pathways post-stroke
Source: Nat Commun. 2019 Aug 6;10:3524. doi: 10.1038/s41467-019-11244-3 (PMC6684621; doi:10.1038/s41467-019-11244-3)
Supplement: Supplementary file 2 — Reporting Summary [file 41467_2019_11244_MOESM2_ESM.pdf]

nature research

Corresponding author(s): Julius P. Dewald

Last updated by author(s): 2019-3-9

Reporting Summary

Nature Research wishes to improve the reproducibility of the work that we publish. This form provides structure for consistency and transparency in reporting. For further information on Nature Research policies, see Authors & Referees and the Editorial Policy Checklist.

Statistics

For all statistical analyses, confirm that the following items are present in the figure legend, table legend, main text, or Methods section.

n/a	Confirmed	
☐	☒	The exact sample size (*n*) for each experimental group/condition, given as a discrete number and unit of measurement
☐	☒	A statement on whether measurements were taken from distinct samples or whether the same sample was measured repeatedly
☐	☒	The statistical test(s) used AND whether they are one- or two-sided<br>*Only common tests should be described solely by name; describe more complex techniques in the Methods section.*
☐	☒	A description of all covariates tested
☐	☒	A description of any assumptions or corrections, such as tests of normality and adjustment for multiple comparisons
☐	☒	A full description of the statistical parameters including central tendency (e.g. means) or other basic estimates (e.g. regression coefficient) AND variation (e.g. standard deviation) or associated estimates of uncertainty (e.g. confidence intervals)
☐	☒	For null hypothesis testing, the test statistic (e.g. *F*, *t*, *r*) with confidence intervals, effect sizes, degrees of freedom and *P* value noted<br>*Give P values as exact values whenever suitable.*
☒	☐	For Bayesian analysis, information on the choice of priors and Markov chain Monte Carlo settings
☒	☐	For hierarchical and complex designs, identification of the appropriate level for tests and full reporting of outcomes
☐	☒	Estimates of effect sizes (e.g. Cohen's *d*, Pearson's *r*), indicating how they were calculated

Our web collection on statistics for biologists contains articles on many of the points above.

Software and code

Policy information about availability of computer code

Data collection	MRI data was collected on Siemens Prisma-fit scanner with the software version VE11C.
Data analysis	Brainstem DTI data was pre-processed using FMRIB software library (FSL) version 5.0.11. Brainstem tract-based spatial statistics analysis for the DTI FA images was carried out using TBSS toolbox in FSL.<br>Spinal cord data preprocessing was done using spinal cord toolbox (SCT; https://www.nitrc.org/projects/sct) version 3.2.2.

For manuscripts utilizing custom algorithms or software that are central to the research but not yet described in published literature, software must be made available to editors/reviewers. We strongly encourage code deposition in a community repository (e.g. GitHub). See the Nature Research guidelines for submitting code & software for further information.

Data

Policy information about availability of data

All manuscripts must include a data availability statement. This statement should provide the following information, where applicable:

- Accession codes, unique identifiers, or web links for publicly available datasets
- A list of figures that have associated raw data
- A description of any restrictions on data availability

The data underlying the findings of this study are available from the corresponding author upon reasonable request.

Field-specific reporting

Please select the one below that is the best fit for your research. If you are not sure, read the appropriate sections before making your selection.

☒ Life sciences ☐ Behavioural & social sciences ☐ Ecological, evolutionary & environmental sciences

For a reference copy of the document with all sections, see nature.com/documents/nr-reporting-summary-flat.pdf

Life sciences study design

All studies must disclose on these points even when the disclosure is negative.

Sample size	The sample size was defined based on the previous literature. Given the size of brainstem and cervical spinal cord and number of voxels included in voxel-wise analysis, we would need at least 20 individuals in each comparing group (stroke vs. controls). We have recruited 36 individuals with chronic unilateral subcortical stroke and 32 age-match gender-match healthy controls to this study.
Data exclusions	5 stroke participants were excluded from the analysis due to brainstem or bilateral lesions. Also, one healthy control was excluded from the analysis due to poor-quality images.
Replication	Our findings were not replication of any previous study, given the novelty of the investigated topic and methods. However, since standard brainstem and spinal cord analysis software are used in this study, our findings can be reproduced by others when using our described imaging protocols and analysis methods.
Randomization	Participants allocation was not random, as we recruited individuals with unilateral stoke and age-match gender-match healthy controls for the group comparison.
Blinding	The blinding was not relevant to this study, as the whole data collection and analysis was done using standard software. Also, no part of data analysis was done visually / manually.

Reporting for specific materials, systems and methods

We require information from authors about some types of materials, experimental systems and methods used in many studies. Here, indicate whether each material, system or method listed is relevant to your study. If you are not sure if a list item applies to your research, read the appropriate section before selecting a response.

Materials & experimental systems

n/a	Involved in the study	
☒	☐	Antibodies
☒	☐	Eukaryotic cell lines
☒	☐	Palaeontology
☒	☐	Animals and other organisms
☐	☒	Human research participants
☒	☐	Clinical data

Methods

n/a	Involved in the study	
☒	☐	ChIP-seq
☒	☐	Flow cytometry
☐	☒	MRI-based neuroimaging

Human research participants

Policy information about studies involving human research participants

Population characteristics	We have recruited individuals with chronic hemiparetic unilateral subcortical stroke. Both ischemic and hemorrhagic stroke with lesions in internal capsule were included. Stroke participants were mildly to severely impaired and all sustained a unilateral brain lesion at least 3 years prior to participation in this study.<br>We also recruited age-match gender-match healthy controls with no neurological or movement impairments for this study.
Recruitment	Individuals with stroke were recruited through the Northwestern Clinical Neuroscience Research Registry, Ischemic Stroke Registry and approved flyers. There was no potential self-selection bias to impact results.
Ethics oversight	The study was approved by the Institutional Review Board of Northwestern University.

Note that full information on the approval of the study protocol must also be provided in the manuscript.

Magnetic resonance imaging

Experimental design

Design type	Brainstem and cervical spinal cord (C2-C5) anatomical and DTI (Diffusion Tensor Imaging) data were collected. No

Design type	functional data (task or resting state) were collected for this study.
Design specifications	Brainstem structural scans duration was about 20 minutes, and the cervical spinal cord structural scans took about 35 minutes.
Behavioral performance measures	No behavioral performance measures were collected.

Acquisition

Imaging type(s)	structural and diffusion
Field strength	3 Testa
Sequence & imaging parameters	Sequence and imaging parameters and reported in the manuscript in full details.<br>Brainstem T1-weighted anatomical scans were acquired with voxel size 0.8 mm isotropic, TR = 9.9 ms, TE = 4.6ms, FOV = 256 mm. Brainstem DWI images were collected with voxel size 1.5 isotropic, TR = 3620 ms, TE = 68.4 ms, matrix size = 150 x 150.<br>Cervical spinal cord T2-W anatomical scans were collected with resolution 0.8 mm isotropic, TR = 1500ms, TE = 100ms, FOV = 256 x 256 mm. cervical spinal cord DWI scans were acquired with 0.8 x 0.8 in-plane resolution, 5 mm slice thickness, TR = 600 ms, TE = 61ms, number of slices = 15.
Area of acquisition	MRI scans were collected from the brainstem and the cervical spinal cord (C2-C5)

Diffusion MRI ☒ Used ☐ Not used

Parameters: Please see above (Sequence and imaging parameters).

Preprocessing

Preprocessing software	Brainstem DTI data was pre-processed using FMRIB software library (FSL) version 5.0.11. Brainstem tract-based spatial statistics analysis for the DTI FA images was carried out using TBSS toolbox in FSL.<br>Spinal cord data preprocessing was done using spinal cord toolbox (SCT; https://www.nitrc.org/projects/sct) version 3.2.2.
Normalization	Brainstem normalization was done using TBSS toolbox in FSL. All subjects FA images were aligned to the standard 1x1x1 mm MNI152 template in FSL, using the non-linear registraion tool (FNIRT).<br>Spinal cord images were nonlinearly registered to the MNI_Poly_AMU template using Spinal Cord Toolbox (SCT).
Normalization template	Please see above.
Noise and artifact removal	All brainstem and spinal cord images were visually inspected and excluded if motion, low signal or artifacts were present.
Volume censoring	Not applicable.

Statistical modeling & inference

Model type and settings	Between group differences in voxel-wise FA (fractional anisotropy) maps were examined using a general linear model (two-sample t-test) design matrix with non-parametric permutation testing, using the Randomize tool in FSL with 50,000 permutations.<br><br>Statistical analysis of spinal cord DTI data was conducted by entering the individual subject FA maps into between group two-sample t-test analysis.
Effect(s) tested	Not applicable.

Specify type of analysis: ☒ Whole brain ☐ ROI-based ☐ Both

Statistic type for inference (See Eklund et al. 2016)	Voxel-wise analysis method were used for both brainstem and spinal cord analyses.<br>Between group differences in voxel-wise FA (fractional anisotropy) maps were examined using a general linear model (two-sample t-test) design matrix with non-parametric permutation testing, using the Randomize tool in FSL with 50,000 permutations.<br><br>Statistical analysis of spinal cord DTI data was conducted by entering the individual subject FA maps into between group two-sample t-test analysis. Voxel-wise p = 0.005 was applied and the cluster-level corrected at p = 0.05.
Correction	For spinal cord analysis, Voxel-wise p = 0.005 was applied and the cluster-level corrected at p = 0.05.

Models & analysis

n/a	Involved in the study
☒	☐ Functional and/or effective connectivity
☒	☐ Graph analysis
☒	☐ Multivariate modeling or predictive analysis
